# Analysis of cumulative risk predictors for persistent stuttering: family perception and amount of speech disruptions

**DOI:** 10.1590/2317-1782/20232022206en

**Published:** 2023-11-13

**Authors:** Julia Biancalana Costa, Fabiola Juste, Ana Paula Ritto, Fernanda Chiarion Sassi, Claudia Regina Furquim de Andrade

**Affiliations:** 1 Divisão de Fonoaudiologia, Faculdade de Medicina, Instituto Central, Hospital das Clínicas, Universidade de São Paulo - USP - São Paulo (SP), Brasil.; 2 Departamento de Fisioterapia, Fonoaudiologia e Terapia Ocupacional, Faculdade de Medicina, Universidade de São Paulo - USP - São Paulo (SP), Brasil.

**Keywords:** Speech, Language and Hearing Sciences, Child, Stuttering, Risk Factors, Indicators

## Abstract

**Purpose:**

To investigate two independent variables considered as two possible predictors of cumulative risk for persistent stuttering: family perception of stuttering and amount of speech disruptions.

**Methods:**

Participants were 452 children, aged 3 to 11:11 years, male and female, divided into 4 groups: group 1 (SCG), composed of 158 children who presented a percentage of stuttered speech disruptions ≥ 3% and family complaint of stuttering; group 2 (SWCG), 42 children who presented percentage of stuttered speech disruptions ≥ 3% and without family complaint of stuttering; group 3 (FCG), 94 children who presented percentage of stuttered speech disruptions ≤ 2. 9% with family complaints of stuttering and group 4 (FWCG), 158 children who presented a percentage of stuttered speech disruptions ≤ 2.9 without family complaints of stuttering.

**Results:**

For the SCG group, there was a significant relationship between family complaints of stuttering and the number of speech disruptions typical of stuttering. In this group, there was a predominance of male children. For the SWCG group, there was no significant relationship between family complaints of stuttering and the number of speech disruptions. For the FCG group, there was no significant relationship between family complaints of stuttering and the number of speech disruptions. For the FWCG group, there was a significant relation between the absence of a family complaint of stuttering and the reduced number of speech disruptions.

**Conclusion:**

The percentage of speech disruptions ≥ 3% is a risk indicator for persistent stuttering. The percentage of speech disruptions ≤ 2.9% associated with syllable and sound repetitions can be a risk indicator for persistent stuttering. Family complaints of syllable and sound repetitions may be a risk indicator for persistent stuttering. Family complaints of stuttering alone should not be considered an indicator of persistent stuttering.

## INTRODUCTION

Persistent stuttering is a neurodevelopmental disorder with a genetic basis. Cellular and neuroimaging studies have already increased our understanding of the biological mechanisms underpinning stuttering, highlighting a significant spatial correspondence between structural anomalies of regional grey matter and the expression of genes associated with energy metabolism. There are signs of cerebral differences between stutterers and fluent individuals, manifest through: grey matter volume; cortical thickness; capacity for diffusion of white matter; metabolic rate, and other neurogenetic parameters. These findings represent a significant scientific advance but still fail to indicate biological mechanisms that disrupt natural speech flow, without the possibility for spontaneous automatic recuperation in response to stuttering symptomatology^(1-9)^.

Fluency disorders during childhood, manifest through symptomatology of speech flow disruptions, are frequently called “infant stuttering”. In epidemiological terms, this “stuttering” affects between 5 to 11% of children of preschool age. It was found that in 75 to 80% of cases, some children spontaneously recover speech fluency, although, for over 20% of children, there was no remission of symptoms - a finding already observed historically in the literature on childhood stuttering. The main question is how to obtain a reliable differential diagnosis to distinguish children with a propensity for spontaneous recovery from children at high risk of persistent stuttering. Children with persistent stuttering, not identified early on, establish an atypical configuration of neural speech-motor networks. Children and their families, not attended to in a timely and competent fashion, tend to delay positive steps for the reduction of negative communicative attitudes and psychical, social, and academic harm, which can negatively affect life outcomes^(10-19)^.

Indicators for high risk of persistent stuttering have been presented in recent publications^(20-24)^, with the following factors found to be relevant: prior family history of persistent stuttering; male sex; speech disruptions characterized by motor effort; symptomatology for more than 12 months and negative family attitudes regarding speech fluency.

This study aimed to relate two independent variables: family perception of stuttering and the amount of speech disruptions. These variables were considered two possible predictors for the cumulative risk of persistent stuttering. The social and scientific relevance of the study is its contribution to clinical and diagnostic decision-making, which will generate a reduction in the impact that persistent stuttering has on the child and their family.

## METHODS

The study is cross-sectional, observational, and retrospective. The data were collected through the analysis of speech sample records from an institutional database. Given the study design, there were no procedures that required signing the Informed Consent form. The project was approved by the Ethics Committee for Analysis of Research Projects (Process 2.001.805).

### Participants

Speech samples from 452 children, with and without stuttering complaints, were analyzed. Their age varied between 3:00 and 11:11 (years:months), of both sexes, without distinction of race or restriction of cultural-socioeconomic level. The speech samples were obtained according to the following criteria: children should be monolingual speakers of Brazilian Portuguese or have acquired (an)other language(s) following the acquisition of Brazilian Portuguese; children should not present oral communication comorbidities (diagnosed or identified through specific screening); children should not present auditory loss of any degree (also diagnosed or identified through specific screening); children should not present any history of neurological and/or degenerative disease. Participants who missed speech sample collection sessions or whose families did not consent to their participation in the study were excluded.

### Speech sample collection

The speech samples were obtained spontaneously, using a stimulation prop in a play situation. Each speech sample (video and audio) was digitally recorded using a high-definition microphone, following the method outlined in the Speech Fluency Profile protocol^(25)^. Spontaneous speech was only interrupted for advice and questions when there was a need to encourage speech production.

### Speech sample analysis

The present study was undertaken with blind analysis of speech samples, to avoid bias, prejudice, poor interpretation of test results, and other information that could affect judgment during transcription. Speech sample analysis was performed by trained speech therapists, who did not participate in the original speech sample collection and who were unaware of the participant’s identity or the presence or absence of stuttering complaints.

The speech samples for this study were analysed and quantified according to the disruptions present in speech flow, differentiated by typology: common or stuttering disruptions. Common disruptions included: hesitations, interjections, revisions, incomplete words, and repeated words, segments, and phrases. Stuttering disruptions included: sound and syllable repetition, blockages, sound and segment intrusions, prolongations, and long pauses in the middle of sentences^(25)^.

To further guarantee the study’s reliability, 15% of the speech samples were submitted to reanalysis by three speech therapist evaluators, experienced in this type of analysis. An 85% agreement level was obtained (k=0.48) indicating high agreement for analysis of results.

### Grouping criteria

**Figure 1:**
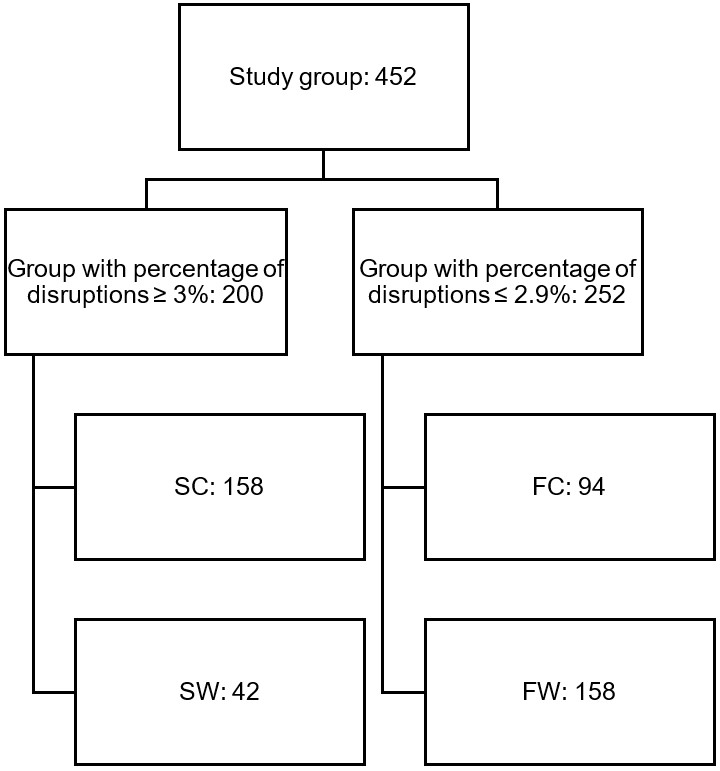
Group flowchart

The first control variable for the study was the rate of stuttered syllables. According to the literature, a consensus exists that speech samples with a percentage of disruptions ≥ 3% are strongly suggestive of stuttering (S)^(16-19)^. The second control variable was family complaints (C) related to stuttering. Based on these variables four distinct groups of children were identified, shown in Figure 1.

**Caption**: SC: stuttering with family complaint; SW: stuttering without family complaint; FC: fluent with family complaint; FW: fluent without family complaint

Group 1 was composed of children with a ≥ 3% percentage of disruptions and family complaints for stuttering (158 participants, or 35.0% of the sample). This group was considered STUTTERING WITH FAMILY COMPLAINT (SC).

Group 2 was composed of children with a ≥ 3% percentage of disruptions and without family complaints for stuttering (42 participants, or 9.2% of the sample). This group was considered STUTTERING WITHOUT FAMILY COMPLAINT (SW).

Group 3 was made up of children with a ≤ 2.9% percentage of disruptions with family complaints for stuttering (94 participants, or 20.8% of the sample). This group was considered FLUENT WITH FAMILY COMPLAINT (FC).

Group 4 was composed of children with a ≤ 2.9% percentage of disruptions without family complaints for stuttering (158 participants, or 35.0% of the sample). This group was considered FLUENT WITHOUT FAMILY COMPLAINT (FW).

### Data analysis

The collected data were submitted to statistical analysis using SPSS software, version 28.0. The data received a descriptive and inferential analysis, comparing the groups (Kruskal-Wallis’s test for quantitative data and Pearson’s chi-squared test for qualitative data, with pairwise post hoc analysis with Bonferroni relation when significant). The tests applied were non-parametric since the data did not present normal distribution, according to the Kolmogorov-Smirnov test. The significance level adopted for all analyses was 5%.

## RESULTS

The groups were compared in relation to their demographic variables: age and sex. A balance between participant ages of the four groups (p=0.45 according to Pearson’s chi-squared test) was observed, with participant ages for all the groups varying between 3 and 11 years, and the median varying between 6.0 and 7.0 years (median; P25-P75 - group SC: 7.0; 4.8-9.0; group SW: 6.5; 4.0-9.0; group FC: 6.0; 4.0-8.0; group FW: 6.5; 4.0-9.0). Regarding sex, there was a significant difference between the groups (p<0.001 according to Pearson’s chi-squared test). The SC group presented 112 male children (70.9%) and 46 female children, (29.1%); the SW group presented 20 male children (47.6%) and 22 female children (52.4%); the FC group presented 62 male children (66.0%) and 32 female children (34.0%); and the FW group presented 68 male children (43.0%) and 90 female children (57.0%). A significant difference was observed for the pairwise comparison between the SC and SW groups, and between the SC and FS groups, with a predominance of male children in the SC group (p=0.040 and p<0.001, respectively, according to a pairwise post hoc analysis with Bonferroni relation). There was also a significant difference between the FC and FW groups, also with a predominance of male children in the FC group (p=0.002 according to pairwise post hoc analysis with Bonferroni relation). Generally, therefore, it was observed that both groups presenting complaints (SC and FC) had more male children in comparison with those groups without complaints (SW and FW).

Regarding the speech fluency evaluation, the groups presented differences (p<0.05 according to the Kruskal-Wallis’s test) for all the variables analysed: total number of common and stuttering disruptions. Pairwise post hoc comparisons with the Bonferroni relation were performed for all variables presenting significant intergroup differences. The SC and SW groups presented no differences for any of the variables cited (p>0.05, according to pairwise post hoc comparison with Bonferroni relation). The other groups were different from one another for most of the variables, except for the total number of common disruptions (p=0.08 in the comparison between SW and FW and p=0.95 in the comparison between FC and FW, according to pairwise post hoc comparison with Bonferroni relation).

Pairwise post hoc comparison with the Bonferroni relation was carried out for all the variables presenting significant intergroup differences in the evaluation for a typology of common disruptions. Notably, the SW and FW groups showed no difference for any variable analysed in relation to common disruptions. The other groups presented differences for most of the variables related to common disruptions, with exceptions, presented in Table 1.

**Table 1:** Pairwise comparison between groups by typology of common disruptions

**Variable**	***p-value***	
Total for common disruptions	SC vs SW: p=0.76	SW vs FC: p<0.001*
	SC vs FC: p<0.001*	SW vs FW: p=0.08
	SC vs FW: p<0.001*	FQ vs FW: p=0.95
Hesitations	SC vs SW: p<0.001*	SW vs FC: p<0.001*
	SC vs FC: p<0.001*	SW vs FW: p=0.19
	SC vs FW: p=0.007*	FC vs FW: p<0.001*
Interjections	SC vs SW: p=0.043*	SW vs FC: p=0.038*
	SC vs FC: p>0.99	SW vs FW: p>0.99
	SC vs FW: p<0.001*	FC vs FW: p<0.001*
Revisions	SC vs SW: p=0.32	SW vs FC: p=0.002*
	SC vs FC: p=0.06	SW vs FW: p>0.99
	SC vs FW: p>0.99	FC vs FW: p=0.006*
Unfinished words	SC vs SW: p>0.99	SW vs FC: p=0.023*
	SC vs FC: p=0.038*	SW vs FW: p>0.99
	SC vs FW: p>0.99	FC vs FW: p=0.001*
Word repetitions	SC vs SW: p<0.001*	SW vs FC: p>0.99
	SC vs FC: p<0.001*	SW vs FW: p>0.99
	SC vs FW: p<0.001*	FC vs FW: p=0.24
Segment repetitions	SC vs SW: p=0.90	SW vs FC: p>0.99
	SC vs FC: p=0.07	SW vs FW: p>0.99
	SC vs FW: p=0.001*	FC vs FW: p>0.99

* significant differences according to pairwise post hoc comparison with Bonferroni relation.

**Caption:** SC: stuttering group with complaint; SW: stuttering group without complaint; FC: fluent group with complaint; FW: fluent group without complaint

The groups were also compared for a typology of stuttering disruptions presented by participants in the speech fluency analysis. The groups presented differences (p<0.05 according to the Kruskal-Wallis’s test) for all the variables analysed. Pairwise post hoc comparisons with the Bonferroni relation were performed for all variables for the evaluation of the typology of stuttering disruptions, with the results presented in Table 2. Notably, the SC group was significantly different from the other groups for nearly all variables analysed, except for the total number of stuttering disruptions (p>0.05 in comparison with SW, according to pairwise post hoc comparison with Bonferroni relation); word ending prolongation (p>0.05 in comparison with SW, according to pairwise post hoc comparison with Bonferroni relation); and pauses (p>0.05 in comparison between the FC and FW groups, according to pairwise post hoc comparison with Bonferroni relation). The SW, FC and FW groups presented no significant differences from one another (p>0.05 according to pairwise post hoc comparison with Bonferroni relation) for most of the variables analysed, with exceptions, presented in Table 2.

**Table 2:** Pairwise comparison between groups by typology of stuttering disruptions.

**Variable**	**p-value**	
Total for stuttering disruptions	SC vs SW: p=0.07	SW vs FC: p<0.001*
	SC vs FC: p<0.001*	SW vs FW: p<0.001*
	SC vs FW: p<0.001*	FC vs FW: p=0.033*
Syllable repetition	SC vs SW: p<0.001*	SW vs FC: p>0.99
	SC vs FC: p<0.001*	SW vs FW: p=0.015*
	SC vs FW: p<0.001*	FC vs FW: p=0.06
Sound repetition	SC vs SW: p<0.001*	SW vs FC: p=0.044*
	SC vs FC: p<0.001*	SW vs FW: p=0.002*
	SC vs FW: p<0.001*	FC vs FW: p>0.99
Prolongations - start and middle of the word	SC vs SW: p<0.001*	SW vs FC: p>0.99
	SC vs FC: p<0.001*	SW vs FW: p>0.99
	SC vs FW: p<0.001*	FC vs FW: p>0.99
Prolongations - word endings	SC vs SW: p>0.99	SW vs FC: p<0.001*
	SC vs FC: p<0.001*	SW vs FW: p<0.001*
	SC vs FW: p<0.001*	FC vs FW: p>0.99
Blockages	SC vs SW: p<0.001*	SW vs FC: p>0.99
	SC vs FC: p<0.001*	SW vs FW: p=0.027*
	SC vs FW: p<0.001*	FC vs FW: p=0.58
Pauses	SC vs SW: p<0.001*	SW vs FC: p<0.001*
	SC vs FC: p=0.434	SW vs FW: p<0.001*
	SC vs FW: p>0.99	FC vs FW: p=0.23
Intrusions	SC vs SW: p<0.001*	SW vs FC: p>0.99
	SC vs FC: p<0.001*	SW vs FW: p>0.99
	SC vs FW: p<0.001*	FC vs FW: p>0.99

* significant differences according to pairwise post hoc comparison with Bonferroni relation.

**Caption:** SC: stuttering group with complaint; SW: stuttering group without complaint; FC: fluent group with complaint; FW: fluent group without complaint;

## DISCUSSION

The research aimed to relate two independent variables: family perception of stuttering and number of speech disruptions. These variables were considered as two possible predictors for the cumulative risk of persistent stuttering.

The definition of predictive indicators, according to NIH (National Institutes of Health)^(26)^ would be the isolation of factors: behavioural and lifestyle; environmental exposure; and innate and/or inherited characteristics. Once the factors are identified, understanding them based on better epidemiological evidence and associating them with those health conditions that can be prevented or treated is the goal for better empirical evidence.

The search for predictive indicators for persistent stuttering allows diagnostic and clinical decision-making that reduces the impact that persistent stuttering has on the child and their family. The consideration of predictive indicators will also facilitate different forms of treatment, providing approaches appropriate to each child’s profile and for their respective families^(17-21)^.

The indicators will also avoid needlessly applying treatments on children presenting a high probability of spontaneous recuperation. Speech therapy treatments should focus on children at high risk of persistent stuttering. It is widely understood that persistent stuttering is associated with negative social and emotional outcomes in quality of life^(20-24)^.

It is generally agreed that, once risk indicators are identified, an approach for the most reliable predictive prognosis for cumulative risk is necessary. In the cumulative risk approach, the greater the number of predictive factors the child presents, the greater the chance of persistent stuttering^(20-24)^.

In recent publications clinical indicators for high risk of persistent stuttering have been presented^(20,24)^, with the following found relevant: family history for persistent stuttering; male sex; speech disruptions characterized by motor effort; symptomatology present for longer than 12 months and negative family attitudes for speech fluency.

The findings from the research undertaken contribute to considerations related to cumulative risk for the identification and prognosis of persistent stuttering. The study data present findings related to two different factors, based on stuttering symptomatology: family perception of stuttering and amount of speech disruptions.

The research results add new findings about indicators for persistent stuttering, including:

1. Observation that both groups presenting complaints (SC and FC) had more male children in comparison with groups without complaints (SW and FW);
2. Children with a percentage of disruptions ≥ 3% perceived by their families as stutterers, can be identified by the following speech disruptions: repeated sounds and syllables; prolongations at the start, middle, or end of works and blockages. For this group, there is a significant relation between family complaints of stuttering and amount of speech disruptions typical of stuttering. In this group, male children predominated;
3. Children with the percentage of disruptions ≥ 3% who are not perceived by their families as stutterers. For this group, although the children present syllable repetition, probably given the lower number of disruptions, family complaints were not reported. This group does not present a significant relation between family complaints of stuttering and the amount of speech disruptions;
4. Children with a percentage of disruptions ≤ 2.9% perceived by their families as stutterers. For this group, the family presented complaints of stuttering, probably, due to speech disruptions of syllable repetition; sound repetitions, and word ending prolongations. For this group, there was no significant relationship between the family complaint and the amount of speech disruptions;
5. Children with the percentage of disruptions ≤ 2.9% who are not perceived by their family as stutterers. The speech disruptions that identify this group are predominantly hesitations, a type of disruption not indicative of stuttering. For this group, there is a significant relation between the absence of family complaints of stuttering and a lower number of speech disruptions.

Based on the findings obtained in the research some study limitations were identified, which will be addressed in future studies, including analysis of the history of family members of participants; differences in the percentages and profiles of disruptions between groups of boys and girls; time from symptom onset and considerations of family attitudes.

## CONCLUSION

In summary:

- The percentage of disruptions ≥ 3% is a risk indicator for persistent stuttering;
- The percentage of disruptions ≤ 2.9% with disruption typology of repetitions can be a risk indicator for persistent stuttering;
- family complaint of disruption of the repetitions type can be a risk indicator for persistent stuttering;
  ● Family complaints of stuttering, in isolation, should not be considered an indicator of persistent stuttering.
