# Supplementary material for: Analysis of cumulative risk predictors for persistent stuttering: family perception and amount of speech disruptions
Source: Codas. 2023 Nov 13;35(6):e20220206. doi: 10.1590/2317-1782/20232022206en (PMC10702700; doi:10.1590/2317-1782/20232022206en)
Supplement: Translation

# Translation: Análise de preditores de risco cumulativo para a gagueira persistente: percepção familiar e quantidade de rupturas da fala

**DOI:** 10.1590/2317-1782/20232022206pt

**Authors:** Julia Biancalana Costa, Fabiola Juste, Ana Paula Ritto, Fernanda Chiarion Sassi, Claudia Regina Furquim de Andrade

**Affiliations:** 1 Divisão de Fonoaudiologia, Faculdade de Medicina, Instituto Central, Hospital das Clínicas, Universidade de São Paulo - USP - São Paulo (SP), Brasil.; 2 Departamento de Fisioterapia, Fonoaudiologia e Terapia Ocupacional, Faculdade de Medicina, Universidade de São Paulo - USP - São Paulo (SP), Brasil.

**Keywords:** Fonoaudiologia, Criança, Gagueira, Fator de Risco, Indicadores

## Abstract

**Objetivo:**

Pesquisar duas variáveis independentes consideradas como possíveis preditores de risco cumulativo para a gagueira persistente (GP): percepção familiar da gagueira e quantidade de rupturas da fala.

**Método:**

Participaram 452 crianças, com idade entre 3 a 11:11 anos, de ambos os gêneros, divididos em 4 grupos: grupo 1 (GGQ), 158 crianças com percentual de rupturas gagas ≥3% e queixa familiar de gagueira; grupo 2 (GGS), 42 crianças com percentual de rupturas gagas ≥3% e sem queixa familiar de gagueira; grupo 3 (FQ), 94 crianças com percentual de rupturas gagas ≤2.9% com queixa familiar de gagueira e grupo 4 (FS), 158 crianças com percentual de rupturas gagas ≤2.9 sem queixa familiar de gagueira.

**Resultados:**

Para o grupo GGQ há relação significante entre a queixa familiar de gagueira e quantidade de rupturas de fala típicas da gagueira e houve predominância de crianças do sexo masculino. Para o grupo GGS não houve relação significante entre a queixa familiar de gagueira e quantidade de rupturas de fala. Para o grupo FQ não houve relação significante entre a queixa familiar de gagueira e quantidade de rupturas de fala. Para o grupo FS houve relação significante entre a ausência de queixa familiar de gagueira e a reduzida quantidade de rupturas de fala.

**Conclusão:**

O percentual de rupturas ≥3% é um indicador de risco para a GP. A queixa familiar de rupturas do tipo repetições pode ser um indicador de risco para a GP. A queixa familiar de gagueira, isoladamente, não deve ser considerada como indicador de GP.

## INTRODUÇÃO

A gagueira persistente é um distúrbio do neurodesenvolvimento com sustentação genética. Os estudos celulares e de neuroimagem já permitem uma maior compreensão dos mecanismos biológicos subjacentes à gagueira indicando uma correspondência espacial significativa entre anomalias estruturais de matéria cinzenta regional e a expressão de genes ligados ao metabolismo energético. Há indicativos de diferenças cerebrais entre pessoas que gaguejam e pessoas fluentes que se manifestam por: volume de matéria cinzenta; espessura cortical; capacidade de difusão da matéria branca; taxa metabólica e outros achados neurogenéticos. Esses achados são um grande avanço científico, mas ainda não indicam o mecanismo biológico que interrompe o fluxo natural da fala, sem possibilidade de recuperação espontânea automática que responde pela sintomatologia da gagueira^(1-9)^.

Os distúrbios da fluência na infância, manifestados pela sintomatologia de rupturas no fluxo na fala, são frequentemente chamados de “gagueira infantil”. Em termos epidemiológicos, essa “gagueira” atinge entre 5 e 11% das crianças em idade pré-escolar. É observado que algumas crianças recuperam a fluência da fala, de forma espontânea, entre 75 e 80% dos casos, embora para mais de 20% das crianças não haja remissão dos sintomas - dado já apresentado historicamente na literatura da gagueira infantil. A condição significativa é como obter um diagnóstico diferencial seguro para diferenciar as crianças propensas à recuperação, daquelas com alto risco da gagueira persistente. As crianças com gagueira persistente, não identificadas precocemente, estabilizam uma configuração atípica das redes motoras neurais da fala. As crianças e suas famílias, não atendidas com rapidez e competência, tendem a atrasar as ações positivas para a redução das atitudes negativas de comunicação e do sofrimento psíquico, social e acadêmico que possam afetar negativamente suas vidas^(10-19)^.

Em publicações recentes foram apresentados indicadores de alto risco para a gagueira persistente^(20-24)^ sendo os confirmadamente relevantes: antecedentes familiares para gagueira persistente; sexo masculino; rupturas de fala caracterizadas pelo esforço motor; sintomatologia há mais de 12 meses e atitudes familiares negativas para a fluência da fala.

O objetivo da pesquisa foi relacionar duas variáveis independentes: percepção familiar da gagueira e quantidade de rupturas da fala. Essas variáveis foram consideradas como dois possíveis preditores de risco cumulativo para a gagueira persistente. A relevância científica e social do estudo é a contribuição para a tomadas de decisões diagnósticas e clínicas, que permitirão a redução do impacto que a gagueira persistente exerce sobre a criança e sua família.

## MÉTODO

A pesquisa se configura como um estudo transversal, observacional, retrospectivo. Os dados foram coletados pela análise de registros de amostras de fala em banco de dados institucional. Pelo desenho da pesquisa não há procedimentos que impliquem em assinatura de Termo de Consentimento Livre e Esclarecido. O projeto foi aprovado pelo Comitê de Ética para Análise de Projetos de Pesquisa do Hospital das Clínicas da Faculdade de Medicina da Universidade de São Paulo ( CAPPesq no 2.001.805).

### Participantes

Foram analisadas as amostras de fala de 452 crianças, com e sem queixa de gagueira. A idade das crianças variou entre 3:0 e 11:11 (anos:meses), de ambos os gêneros, sem distinção de raça e sem restrição de nível socioeconômico-cultural. As amostras de fala foram obtidas seguindo os critérios: as crianças deveriam ser falantes monolíngue do Português Brasileiro ou terem adquirido outra(s) língua(s) após a aquisição do Português Brasileiro; as crianças não poderiam apresentar comorbidades da comunicação oral (diagnosticadas ou identificadas em teste de triagem específica); as crianças não poderiam apresentar perda auditiva de qualquer grau (também diagnosticada ou identificada em teste de triagem específica); as crianças não poderiam apresentar histórico de doenças neurológicas e/ou degenerativas. Foram excluídos do estudo os participantes que faltaram às sessões de coleta das amostras da fala ou cuja família não consentiu com a participação no estudo.

### Coleta das amostras de fala

As amostras de fala foram obtidas em situação de fala espontânea, a partir de uma figura estímulo ou em situação de brincadeira. Cada amostra de fala (vídeo e áudio) foi gravada digitalmente com microfone de alta definição, e em conformidade com o método descrito no protocolo do Perfil da Fluência de Fala^(25)^. A fala espontânea só foi interrompida por comentários e perguntas caso houvesse a necessidade de incentivar a produção do discurso.

### Análise das amostras de fala

O presente estudo foi realizado com cegamento de análise das amostras de fala, para evitar vieses, preconceitos, má interpretação dos resultados do teste e outras informações que pudessem afetar o julgamento durante a transcrição. As análises das amostras de fala foram realizadas por fonoaudiólogos qualificados, que não participaram da coleta das amostras originais de fala e que não estavam cientes da identificação dos participantes e da presença ou não de queixa de gagueira.

As amostras de fala, para esse estudo, foram analisadas e quantificadas segundo as rupturas apresentadas no fluxo da fala, diferenciadas pela tipologia: rupturas comuns ou gagas. As rupturas comuns foram: hesitações, interjeições, revisões, palavras incompletas e repetições de palavras, segmentos e frases. As rupturas gagas foram: repetições de sons e sílabas, bloqueios, intrusões de sons e segmentos, prolongamentos e pausas longas no meio de sentenças^(25)^.

Para ampliar a confiabilidade do estudo, um total de 15% das amostras de fala foram submetidas à reanálise de três juízes, fonoaudiólogos, com experiência nesse tipo de análise. Foi obtido o nível de concordância de 85% (k=0,48) indicando alta concordância na análise dos resultados.

### Critérios para os agrupamentos

A primeira variável de controle do estudo foi a taxa de sílabas gaguejadas. Conforme dados da literatura há consenso que amostras de fala com percentual de rupturas ≥ 3% são amostras fortemente sugestivas de gagueira (GG)^(16-19)^. A segunda variável de controle foi a queixa familiar (Q) relacionada à gagueira. A partir dessas variáveis foram identificados quatro grupos distintos de crianças, conforme Figura 1.

**Figura 1:** Fluxograma do agrupamento

**Legenda:** GGQ: gagueira com queixa familiar; GGS: gagueira sem queixa familiar; FQ: fluente com queixa familiar; FS: fluente sem queixa familiar

O Grupo 1 foi composto por crianças com percentual de rupturas ≥ 3% e queixa familiar de gagueira (158 participantes, ou 35,0% da amostra). Esse grupo foi considerado GAGUEIRA COM QUEIXA FAMILIAR (GGQ).

O Grupo 2 foi composto por crianças com percentual de rupturas ≥ 3% e sem queixa familiar de gagueira (42 participantes, ou 9,2% da amostra). Esse grupo foi considerado GAGUEIRA SEM QUEIXA FAMILIAR (GGS).

O Grupo 3 foi composto por crianças com percentual de rupturas ≤ 2.9% com queixa familiar de gagueira (94 participantes, ou 20,8% da amostra). Esse grupo foi considerado FLUENTE COM QUEIXA FAMILIAR (FQ).

O grupo 4 foi composto por crianças com percentual de rupturas ≤ 2.9% sem queixa familiar de gagueira (158 participantes, ou 35,0% da amostra). Esse grupo foi considerado FLUENTE COM QUEIXA FAMILIAR (FS).

### Análise de dados

Os dados coletados foram submetidos à análise estatística no software SPSS versão 28.0. Os dados receberam análise descritiva e inferencial, comparando os grupos (teste de Kruskal-Wallis para os dados quantitativos e teste Qui-Quadrado de Pearson para os dados qualitativos, com análise pairwise post hoc com relação de Bonferroni, se significativo). Os testes aplicados foram não-paramétricos pois os dados não apresentaram distribuição normal, de acordo com o teste de Kolmogorov-Smirnov. O nível de significância adotado em todas as análises foi de 5%.

## RESULTADOS

Os grupos foram comparados com relação às suas variáveis demográficas: idade e gênero. Observou-se que houve equilíbrio entre as idades dos participantes dos quatro grupos (p=0,45 de acordo com o teste Qui-quadrado de Pearson), com as idades dos participantes de todos os grupos variando entre 3 e 11 anos, e a mediana variando entre 6,0 e 7,0 anos (mediana; P25-P75 - grupo GGQ: 7,0; 4,8-9,0; grupo GGS: 6,5; 4,0-9,0; grupo FQ: 6,0; 4,0-8,0; grupo FS: 6,5; 4,0-9,0). Quanto ao gênero, houve diferença significativa entre os grupos (p<0,001 de acordo com o teste Qui-quadrado de Pearson). O grupo GGQ apresentou 112 crianças do sexo masculino (70,9%) e 46 do sexo feminino, (29,1%); o grupo GGS apresentou 20 crianças do sexo masculino (47,6%) e 22 do sexo feminino (52,4%); o grupo FQ apresentou 62 crianças do sexo masculino (66,0%) e 32 do sexo feminino (34,0%); e o grupo FS apresentou 68 crianças do sexo masculino (43,0%) e 90 do sexo feminino (57,0%). Observa-se diferença significativa na comparação de pares entre os grupos GGQ e GGS, e entre os grupos GGQ e FS, com a predominância de crianças do sexo masculino no grupo GGQ (p=0,040 e p<0,001, respectivamente, de acordo com a comparação *pairwise post hoc* com relação de Bonferroni). Também houve diferença significativa entre os grupos FQ e FS, também com a predominância de crianças do sexo masculino no grupo FQ (p=0,002 de acordo com a comparação *pairwise post hoc* com relação de Bonferroni). De forma geral, portanto, observa-se que ambos os grupos com queixa (GGQ e FQ) têm mais crianças do sexo masculino quando comparados aos grupos sem queixa (GGS e FS).

Com relação à avaliação da fluência de fala, os grupos se diferenciaram (p<0,05 de acordo com o teste de Kruskal-Wallis) em todas as variáveis analisadas: número total de rupturas comuns e número total de rupturas gagas. Foram realizadas comparações *pairwise post hoc* com relação de Bonferroni para todas as variáveis com diferença significativa intergrupos. Os grupos GGQ e GGS não se diferenciaram em nenhuma das variáveis citadas (p>0,05, de acordo com a comparação *pairwise post hoc* com relação de Bonferroni). Já os outros grupos diferenciam-se entre si para a maioria das variáveis, exceto número total de rupturas comuns (p=0,08 na comparação entre GGS e FS e p=0,95 na comparação entre FQ e FS, de acordo com a comparação *pairwise post hoc* com relação de Bonferroni).

Foram realizadas comparações *pairwise post hoc* com relação de Bonferroni para todas as variáveis com diferença significativa intergrupos na avaliação da tipologia das rupturas comuns. Nota-se que os grupos GGS e FS não se diferenciaram em nenhuma das variáveis analisadas referentes às rupturas comuns. Já os outros grupos diferenciam-se entre si para a maioria das variáveis referentes às rupturas comuns, com exceções, conforme Tabela 1.

**Tabela 1:** Comparação de pares entre os grupos quanto à tipologia das rupturas comuns

**Variável**	***p-value***	
Total de rupturas comuns	GGQ vs GGS: p=0,76	GGS vs FQ: p<0,001*
	GGQ vs FQ: p<0,001*	GGS vs FS: p=0,08
	GGQ vs FS: p<0,001*	FQ vs FS: p=0,95
Hesitações	GGQ vs GGS: p<0,001*	GGS vs FQ: p<0,001*
	GGQ vs FQ: p<0,001*	GGS vs FS: p=0,19
	GGQ vs FS: p=0,007*	FQ vs FS: p<0,001*
Interjeições	GGQ vs GGS: p=0,043*	GGS vs FQ: p=0,038*
	GGQ vs FQ: p>0,99	GGS vs FS: p>0,99
	GGQ vs FS: p<0,001*	FQ vs FS: p<0,001*
Revisões	GGQ vs GGS: p=0,32	GGS vs FQ: p=0,002*
	GGQ vs FQ: p=0,06	GGS vs FS: p>0,99
	GGQ vs FS: p>0,99	FQ vs FS: p=0,006*
Palavras não terminadas	GGQ vs GGS: p>0,99	GGS vs FQ: p=0,023*
	GGQ vs FQ: p=0,038*	GGS vs FS: p>0,99
	GGQ vs FS: p>0,99	FQ vs FS: p=0,001*
Repetições de palavras	GGQ vs GGS: p<0,001*	GGS vs FQ: p>0,99
	GGQ vs FQ: p<0,001*	GGS vs FS: p>0,99
	*GGQ vs FS: p<0,001* *	FQ vs FS: p=0,24
Repetições de segmentos	GGQ vs GGS: p=0,90	GGS vs FQ: p>0,99
	GGQ vs FQ: p=0,07	GGS vs FS: p>0,99
	GGQ vs FS: p=0,001*	FQ vs FS: p>0,99

* diferença significativa de acordo com a comparação *pairwise post hoc* com relação de Bonferroni

**Legenda:** GGQ: grupo gagueira com queixa; GGS: grupo gagueira sem queixa; FQ: grupo fluente com queixa; FS: grupo fluente sem queixa

Os grupos foram comparados ainda com relação à tipologia das rupturas gagas apresentadas pelos participantes na avaliação da fluência de fala. Os grupos se diferenciaram (p<0,05 de acordo com o teste de Kruskal-Wallis) em todas as variáveis analisadas. Foram realizadas comparações *pairwise post hoc* com relação de Bonferroni para todas as variáveis da avaliação da tipologia das rupturas gagas, e os resultados encontram-se disponíveis na Tabela 2. Nota-se que o grupo GGQ diferencia-se significativamente dos outros grupos para quase todas as variáveis analisadas, exceto número total de rupturas gagas (p>0,05 na comparação com GGS, de acordo com a comparação *pairwise post hoc* com relação de Bonferroni); prolongamento em fim de palavra (p>0,05 na comparação com GGS, de acordo com a comparação *pairwise post hoc* com relação de Bonferroni); e pausa (p>0,05 na comparação com FQ e FS, de acordo com a comparação *pairwise post hoc* com relação de Bonferroni). Já os grupos GGS, FQ e FS não apresentam diferenças significativas entre si (p>0,05, de acordo com a comparação *pairwise post hoc* com relação de Bonferroni) para a maioria das variáveis analisadas, com exceções, conforme Tabela 2.

**Tabela 2:** Comparação de pares entre os grupos quanto à tipologia das rupturas gagas

**Variável**	***p-value***	
Total de rupturas gagas	GGQ vs GGS: p=0,07	GGS vs FQ: p<0,001*
	GGQ vs FQ: p<0,001*	GGS vs FS: p<0,001*
	GGQ vs FS: p<0,001*	FQ vs FS: p=0,033*
Repetições de sílabas	GGQ vs GGS: p<0,001*	GGS vs FQ: p>0,99
	GGQ vs FQ: p<0,001*	GGS vs FS: p=0,015*
	GGQ vs FS: p<0,001*	FQ vs FS: p=0,06
Repetições de sons	GGQ vs GGS: p<0,001*	GGS vs FQ: p=0,044*
	GGQ vs FQ: p<0,001*	GGS vs FS: p=0,002*
	GGQ vs FS: p<0,001*	FQ vs FS: p>0,99
Prolongamentos - início e meio da palavra	GGQ vs GGS: p<0,001*	GGS vs FQ: p>0,99
	GGQ vs FQ: p<0,001*	GGS vs FS: p>0,99
	GGQ vs FS: p<0,001*	FQ vs FS: p>0,99
Prolongamentos - fim da palavra	GGQ vs GGS: p>0,99	GGS vs FQ: p<0,001*
	GGQ vs FQ: p<0,001*	GGS vs FS: p<0,001*
	GGQ vs FS: p<0,001*	FQ vs FS: p>0,99
Bloqueios	GGQ vs GGS: p<0,001*	GGS vs FQ: p>0,99
	GGQ vs FQ: p<0,001*	GGS vs FS: p=0,027*
	GGQ vs FS: p<0,001*	FQ vs FS: p=0,58
Pausas	GGQ vs GGS: p<0,001*	GGS vs FQ: p<0,001*
	GGQ vs FQ: p=0,434	*GGS vs FS: p<0,001* *
	GGQ vs FS: p>0,99	FQ vs FS: p=0,23
Intrusões	GGQ vs GGS: p<0,001*	GGS vs FQ: p>0,99
	GGQ vs FQ: p<0,001*	GGS vs FS: p>0,99
	GGQ vs FS: p<0,001*	FQ vs FS: p>0,99

* diferença significativa de acordo com a comparação *pairwise post hoc* com relação de Bonferroni

**Legenda:** GGQ: grupo gagueira com queixa; GGS: grupo gagueira sem queixa; FQ: grupo fluente com queixa; FS: grupo fluente sem queixa

## DISCUSSÃO

O objetivo da pesquisa foi relacionar duas variáveis independentes: percepção familiar da gagueira e quantidade de rupturas da fala. Essas variáveis foram consideradas como dois possíveis preditores de risco cumulativo para a gagueira persistente.

A definição de indicadores preditivos, segundo a NIH (*National Institutes of Health*)^(26)^ seria o isolamento de fatores: comportamentais ou de estilo de vida; de exposição ambiental; de características inatas e/ou herdadas. Uma vez isolados os fatores, entendê-los com base em evidências epidemiológicas e, associá-los às condições de saúde que possam ser prevenidas ou tratadas precocemente é a meta para melhores evidências empíricas.

A busca por indicadores preditivos para a gagueira persistente permitirá que sejam tomadas medidas diagnósticas e clínicas que reduzirão o impacto que a gagueira persistente exerce sobre a criança e sua família. A consideração dos indicadores preditivos permitirá que sejam oferecidos diferentes tipos de tratamento, com abordagens apropriadas para o perfil de cada criança e sua família^(17-21)^.

Os indicadores também permitirão que não sejam aplicados tratamentos desnecessários às crianças com alta probabilidade de recuperação espontânea. O tratamento fonoaudiológico deve ser direcionado às crianças com alto risco para a gagueira persistente. É amplamente reconhecido que a persistência gagueira está associada aos desfechos sociais, emocionais e impacto negativo na qualidade de vida^(20-24)^.

Há consenso que, uma vez identificados os indicadores de risco, é necessária uma abordagem sobre o risco cumulativo para uma previsão prognóstica mais segura. Na abordagem de risco cumulativo, quanto maior o número de fatores preditivos a criança apresentar, maior a chance de gagueira persistente^(20-24)^.

Em publicações recentes foram apresentados indicadores clínicos de alto risco para a gagueira persistente^(20,24)^ sendo os confirmadamente relevantes: antecedentes familiares para gagueira persistente; sexo masculino; rupturas de fala caracterizadas pelo esforço motor; sintomatologia presente há mais de 12 meses e atitudes familiares negativas para a fluência da fala.

Os achados da pesquisa realizada contribuem para as considerações sobre os riscos cumulativos para a identificação e prognóstico da gagueira persistente. Os dados da pesquisa apresentam considerações sobre dois diferentes fatores, com base na sintomatologia da gagueira: percepção familiar da gagueira e quantidade de rupturas da fala.

Os resultados da pesquisa acrescentam novas considerações sobre os indicadores de persistência da gagueira, sendo:

1. Foi observado que ambos os grupos com queixa (GGQ e FQ) têm mais crianças do sexo masculino quando comparados aos grupos sem queixa (GGS e FS);
2. Crianças com percentual de rupturas ≥ 3% que são percebidos pelas famílias como crianças com gagueira. As rupturas de fala que identificam esse grupo são: repetições de sons e sílabas; prolongamentos em início, meio ou fim das palavras e bloqueios. Para esse grupo há relação significante entre a queixa familiar de gagueira e quantidade de rupturas de fala típicas da gagueira. Nesse grupo há predominância de crianças do sexo masculino;
3. Crianças com percentual de rupturas ≥ 3% que não são percebidas pela família como crianças com gagueira. Para esse grupo, embora as crianças apresentem repetições de sílabas, provavelmente pela menor quantidade dessas rupturas, não há queixa familiar. Esse grupo não apresenta relação significante entre a queixa familiar de gagueira e quantidade de rupturas de fala;
4. Crianças com percentual de rupturas ≤ 2.9% que são percebidas pela família como crianças com gagueira. Para esse grupo, a família apresenta queixa de gagueira, provavelmente, em decorrência das rupturas de fala de: repetições de sílabas; repetições de sons e prolongamentos no final das palavras. Para esse grupo não há relação significante entre a queixa familiar de gagueira e quantidade de rupturas de fala;
5. Crianças com percentual de rupturas ≤ 2.9% que não são percebidas pela família como crianças com gagueira. As rupturas de fala que identificam esse grupo são predominantemente as hesitações, tipo de ruptura não indicativa de gagueira. Para esse grupo há relação significante entre a ausência de queixa familiar de gagueira e a reduzida quantidade de rupturas de fala.

A partir dos resultados obtidos na pesquisa foram identificadas algumas limitações do estudo que serão abordadas em futuros estudos como: análise dos antecedentes familiares dos participantes; diferenças nos percentuais e perfis das rupturas em grupos de meninos e meninas; tempo de início dos sintomas e considerações sobre as atitudes familiares.

## CONCLUSÃO

Em síntese:

- o percentual de rupturas ≥ 3% é um indicador de risco para a gagueira persistente;
- o percentual de rupturas ≤ 2.9% com tipologia de rupturas de repetições pode ser um indicador de risco para a gagueira persistente;
- a queixa familiar de rupturas do tipo repetições pode ser um indicador de risco para a gagueira persistente;
  ● a queixa familiar de gagueira, isoladamente, não deve ser considerada como indicador de gagueira persistente.
